# 1,2-Bis(3-phen­oxy­benzyl­idene)hydrazine

**DOI:** 10.1107/S1600536811052469

**Published:** 2011-12-10

**Authors:** Jerry P. Jasinski, James A. Golen, A. S. Praveen, B. Narayana, H. S. Yathirajan

**Affiliations:** a Department of Chemistry, Keene State College, 229 Main Street, Keene, NH 03435-2001, USA; b Department of Studies in Chemistry, University of Mysore, Manasagangotri, Mysore 570 006, India; c Department of Studies in Chemistry, Mangalore University, Mangalagangotri, 574 199, India

## Abstract

Mol­ecules of the title compound, C_26_H_20_N_2_O_2_, reside on crystallographic centres of inversion located at the mid-point of the N—N bond. The benzyl­idene ring is essentially coplanar with the central hydrazine group, with an inter­planar angle of 4.5 (2)°, whereas the phenyl ring is oriented at 34.0 (3)° with respect to the mean plane of the central 1,2-dibenzyl­idenehydrazine group. In the crystal, C—H⋯π(arene)-ring inter­actions link mol­ecules about inversion centres.

## Related literature

For the biological activity of Schiff bases, see: Aydogan *et al.* (2001 ▶); Desai *et al.* (2001 ▶); El-masry *et al.* (2000 ▶); Hodnett & Dunn (1970 ▶); Kundu *et al.* (2005 ▶); Pandeya *et al.* (1999 ▶); Singh & Dash (1988 ▶); Taggi *et al.* (2002 ▶); Xu *et al.* (1997 ▶); For crystallography and coordination chemistry of compounds containing the azine functionality or a diimine linkage, see: Xu *et al.* (1997 ▶); Kundu *et al.* (2005 ▶); For related structures, see: Liu *et al.* (2007 ▶); Odabaşoğlu *et al.* (2007 ▶); Zhang & Zheng (2008 ▶); Zheng *et al.* (2005*a* ▶,*b* ▶). For standard bond lengths, see Allen *et al.* (1987 ▶).
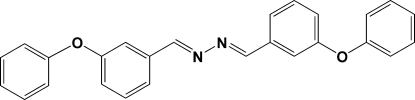


## Experimental

### 

#### Crystal data

C_26_H_20_N_2_O_2_
*M* *_r_* = 392.44
Monoclinic, 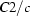

*a* = 23.6271 (16) Å
*b* = 11.2942 (6) Å
*c* = 8.2359 (7) Å
β = 109.538 (8)°
*V* = 2071.2 (3) Å^3^
*Z* = 4
Mo *K*α radiation
μ = 0.08 mm^−1^
*T* = 170 K
0.44 × 0.20 × 0.06 mm

#### Data collection

Oxford Diffraction Xcalibur Eos Gemini diffractometer
Absorption correction: multi-scan (*CrysAlis RED*; Oxford Diffraction, 2010 ▶) *T*_min_ = 0.966, *T*_max_ = 0.995
4228 measured reflections
2341 independent reflections
1643 reflections with *I* > 2σ(*I*)
*R*_int_ = 0.023

#### Refinement

*R*[*F* ^2^ > 2σ(*F* ^2^)] = 0.050
*wR*(*F* ^2^) = 0.144
*S* = 1.05
2341 reflections
136 parameters
H-atom parameters constrained
Δρ_max_ = 0.14 e Å^−3^
Δρ_min_ = −0.19 e Å^−3^

### 

Data collection: *CrysAlis PRO* (Oxford Diffraction, 2010 ▶); cell refinement: *CrysAlis PRO*; data reduction: *CrysAlis RED* (Oxford Diffraction, 2010 ▶); program(s) used to solve structure: *SHELXS97* (Sheldrick, 2008 ▶); program(s) used to refine structure: *SHELXL97* (Sheldrick, 2008 ▶); molecular graphics: *SHELXTL* (Sheldrick, 2008 ▶); software used to prepare material for publication: *SHELXTL*.

## Supplementary Material

Crystal structure: contains datablock(s) global, I. DOI: 10.1107/S1600536811052469/gg2067sup1.cif

Structure factors: contains datablock(s) I. DOI: 10.1107/S1600536811052469/gg2067Isup2.hkl

Supplementary material file. DOI: 10.1107/S1600536811052469/gg2067Isup3.cml

Additional supplementary materials:  crystallographic information; 3D view; checkCIF report

## Figures and Tables

**Table 1:** Hydrogen-bond geometry (Å, °) *Cg* is the centroid of the C7–C12 benzyl­idene ring.

*D*—H⋯*A*	*D*—H	H⋯*A*	*D*⋯*A*	*D*—H⋯*A*
C5—H5*A*⋯*Cg*^i^	0.93	2.68	3.5947 (17)	167

Symmetry code: (i) 
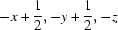
.

**Fig. 1.**
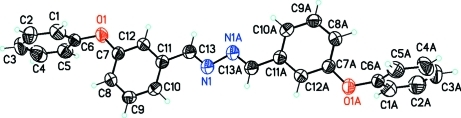
Molecular structure of the title compound showing the atom labeling scheme and 50% probability displacement ellipsoids.

**Fig. 2.**
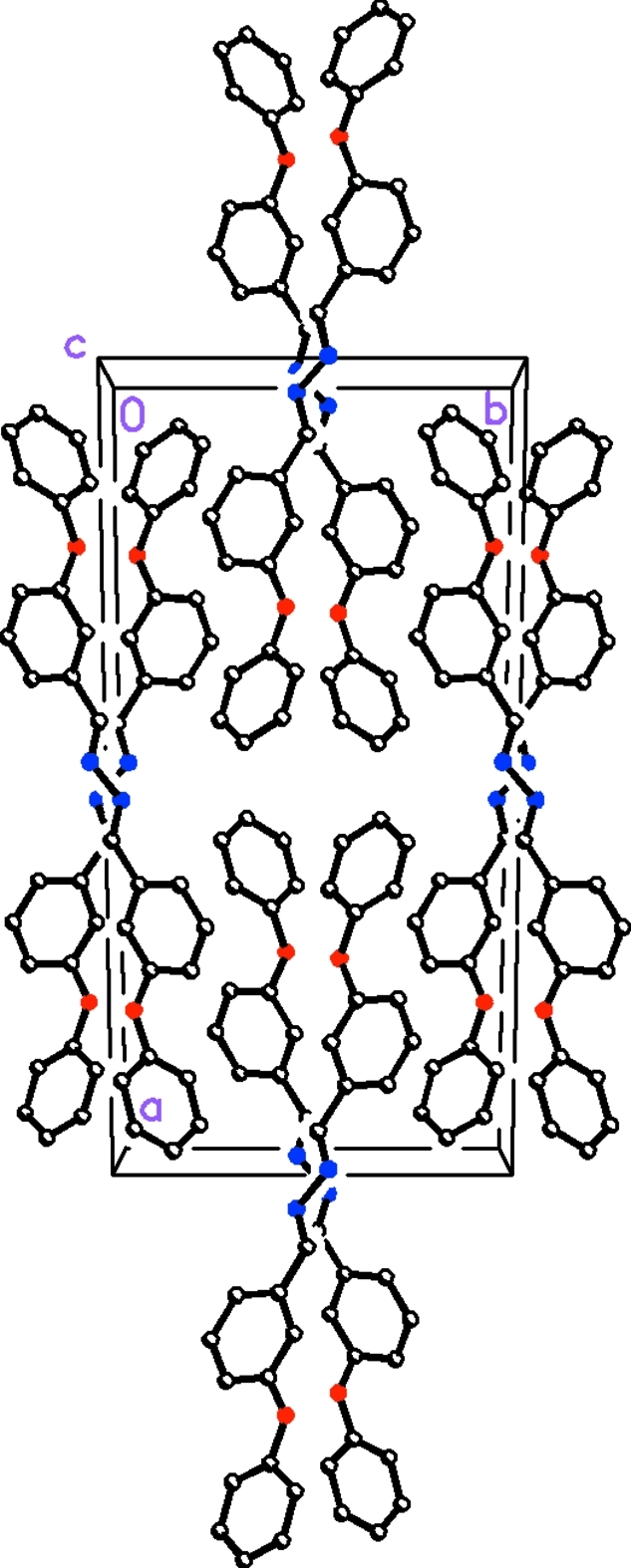
Packing diagram of the title compound viewed along the c axis. The H atoms have been removed for clarity.

## supplementary crystallographic information

### Comment

Schiff bases are known to exhibit biological activity such as antimicrobial (El-masry *et al.*, 2000 & Pandeya *et al.*, 1999), antifungal (Singh & Dash, 1988), antitumor (Hodnett & Dunn, 1970; Desai *et al.*, 2001) and as herbicides. Moreover, Schiff bases are used as substrates in the preparation of number of industrial and biologically active compounds *via* ring closure, cycloaddition and replacement reactions. Schiff bases have also been employed as ligands for complexation of metal ions (Aydogan  *et al.*,  2001). On the industrial scale, they have wide range of applications such as dyes and pigments (Taggi  *et al.*,  2002).  Compounds containing an azine functionality or a diimine linkage have been investigated in terms of their crystallography and coordination chemistry (Xu *et al.*, 1997; Kundu *et al.*, 2005).

The crystal structures of some Schiff base hydrazines, *viz.*, 4-fluorobenzaldehyde[(E)-4-fluorobenzylidene]hydrazone (Odabaşoğlu *et al.*, 2007), N,N'-bis(3-nitrobenzylidene)hydrazine (Zheng *et al.*, 2005*a*), N,N'-Bis(4-chlorobenzylidene)hydrazine (Zheng *et al.*, 2005*b*), 1,2-bis(2-chlorobenzylidene)hydrazine (Zhang &  Zheng, 2008), N,N'-Bis(4-hydroxybenzylidene)hydrazine (Liu *et al.*, 2007) have been reported. In view of the importance of Schiff base hydrazines, the crystal structure of title compound (I) is reported herein.

The complete molecule of the title compound, C_26_H_20_N_2_O_2_, is generated by the application of a centre of inversion (Fig. 1). The benzylidene ring is essentially coplanar with the central hydrazine group [dihedral angle = 4.5 (2)°]  The dihedral angle between the mean plane of the 1,2-bis(benzylidene) hydrazine group and the two parallel phenyl rings is 34.0 (3)°.  Weak C—H···Cg π-ring intermolecular interactions are observed (Table 1) providing some crystal packing stability (Fig. 2).

### Experimental

A mixture of 3-phenoxybenzaldehyde (0.02 mol) and hydrazine hydrate (0.012 mol) was refluxed in 15 ml of absolute alcohol containing 2 drops of sulfuric acid, for about 3 hours. On cooling, the resulting solid was filtered and dried. Single crystals were grown from DMF (dimethylformamide) by the slow evaporation method. Yield: 86%. (m.p.: 404 K).

### Refinement

All of the H atoms were placed in their calculated positions and then refined using the riding model with Atom—H lengths of 0.93Å (C–H). Isotropic displacement parameters for these atoms were set to 1.19-1.20 (CH) times *U*_eq_ of the parent atom.

### Crystal data

**Table:**

C_26_H_20_N_2_O_2_	*F*(000) = 824
*M**_r_* = 392.44	*D*_x_ = 1.259 Mg m^−^^3^
Monoclinic, *C*2/*c*	Mo *K*α radiation, λ = 0.71073 Å
Hall symbol: -C 2yc	Cell parameters from 1146 reflections
*a* = 23.6271 (16) Å	θ = 3.1–28.6°
*b* = 11.2942 (6) Å	µ = 0.08 mm^−^^1^
*c* = 8.2359 (7) Å	*T* = 170 K
β = 109.538 (8)°	Plate, yellow
*V* = 2071.2 (3) Å^3^	0.44 × 0.20 × 0.06 mm
*Z* = 4	

### Data collection

**Table:**

Oxford Diffraction Xcalibur Eos Gemini diffractometer	2341 independent reflections
Radiation source: Enhance (Mo) X-ray Source	1643 reflections with *I* > 2σ(*I*)
graphite	*R*_int_ = 0.023
Detector resolution: 16.1500 pixels mm^-1^	θ_max_ = 28.7°, θ_min_ = 3.1°
ω scans	*h* = −31→19
Absorption correction: multi-scan (*CrysAlis RED*; Oxford Diffraction, 2010)	*k* = −12→14
*T*_min_ = 0.966, *T*_max_ = 0.995	*l* = −10→10
4228 measured reflections	

### Refinement

**Table:**

Refinement on *F*^2^	Primary atom site location: structure-invariant direct methods
Least-squares matrix: full	Secondary atom site location: difference Fourier map
*R*[*F*^2^ > 2σ(*F*^2^)] = 0.050	Hydrogen site location: inferred from neighbouring sites
*wR*(*F*^2^) = 0.144	H-atom parameters constrained
*S* = 1.05	*w* = 1/[σ^2^(*F*_o_^2^) + (0.0591*P*)^2^ + 0.4457*P*] where *P* = (*F*_o_^2^ + 2*F*_c_^2^)/3
2341 reflections	(Δ/σ)_max_ < 0.001
136 parameters	Δρ_max_ = 0.14 e Å^−^^3^
0 restraints	Δρ_min_ = −0.19 e Å^−^^3^

### Special details

**Table:**

Geometry. All esds (except the esd in the dihedral angle between two l.s. planes) are estimated using the full covariance matrix. The cell esds are taken into account individually in the estimation of esds in distances, angles and torsion angles; correlations between esds in cell parameters are only used when they are defined by crystal symmetry. An approximate (isotropic) treatment of cell esds is used for estimating esds involving l.s. planes.
Refinement. Refinement of *F*^2^ against ALL reflections. The weighted *R*-factor *wR* and goodness of fit *S* are based on *F*^2^, conventional *R*-factors *R* are based on *F*, with *F* set to zero for negative *F*^2^. The threshold expression of *F*^2^ > σ(*F*^2^) is used only for calculating *R*-factors(gt) *etc*. and is not relevant to the choice of reflections for refinement. *R*-factors based on *F*^2^ are statistically about twice as large as those based on *F*, and *R*- factors based on ALL data will be even larger.

### Fractional atomic coordinates and isotropic or equivalent isotropic displacement parameters (Å^2^)

**Table:**

	*x*	*y*	*z*	*U*_iso_*/*U*_eq_	
O1	0.28415 (5)	0.56492 (12)	0.14723 (17)	0.0634 (4)	
N1	0.02329 (6)	0.53855 (12)	0.04610 (19)	0.0533 (4)	
C1	0.36654 (9)	0.54126 (16)	0.4051 (2)	0.0582 (5)	
H1B	0.3467	0.4787	0.4361	0.070*	
C2	0.42338 (10)	0.5731 (2)	0.5077 (3)	0.0769 (7)	
H2A	0.4421	0.5321	0.6096	0.092*	
C3	0.45254 (9)	0.6644 (2)	0.4612 (3)	0.0818 (7)	
H3A	0.4911	0.6854	0.5310	0.098*	
C4	0.42510 (10)	0.7249 (2)	0.3120 (3)	0.0760 (7)	
H4A	0.4450	0.7870	0.2802	0.091*	
C5	0.36825 (9)	0.69433 (15)	0.2091 (2)	0.0584 (5)	
H5A	0.3493	0.7355	0.1076	0.070*	
C6	0.33985 (7)	0.60286 (14)	0.2574 (2)	0.0432 (4)	
C7	0.23306 (7)	0.60721 (15)	0.1737 (2)	0.0442 (4)	
C8	0.23209 (7)	0.70730 (14)	0.2691 (2)	0.0483 (4)	
H8A	0.2672	0.7497	0.3218	0.058*	
C9	0.17840 (8)	0.74360 (15)	0.2851 (2)	0.0491 (4)	
H9A	0.1776	0.8111	0.3491	0.059*	
C10	0.12623 (7)	0.68200 (14)	0.2084 (2)	0.0462 (4)	
H10A	0.0905	0.7071	0.2213	0.055*	
C11	0.12717 (7)	0.58123 (13)	0.1108 (2)	0.0416 (4)	
C12	0.18101 (7)	0.54480 (14)	0.0936 (2)	0.0441 (4)	
H12A	0.1820	0.4783	0.0281	0.053*	
C13	0.07271 (7)	0.51346 (15)	0.0257 (2)	0.0469 (4)	
H13A	0.0743	0.4504	−0.0452	0.056*	

### Atomic displacement parameters (Å^2^)

**Table:**

	*U*^11^	*U*^22^	*U*^33^	*U*^12^	*U*^13^	*U*^23^
O1	0.0376 (6)	0.0815 (9)	0.0687 (9)	−0.0038 (6)	0.0148 (6)	−0.0324 (7)
N1	0.0396 (8)	0.0516 (9)	0.0670 (10)	−0.0047 (6)	0.0156 (7)	−0.0052 (7)
C1	0.0663 (12)	0.0553 (11)	0.0548 (11)	−0.0063 (9)	0.0226 (9)	0.0036 (9)
C2	0.0680 (14)	0.0838 (16)	0.0605 (13)	0.0133 (13)	−0.0029 (11)	0.0005 (11)
C3	0.0425 (11)	0.0966 (18)	0.0949 (18)	−0.0106 (12)	0.0079 (11)	−0.0309 (15)
C4	0.0707 (14)	0.0690 (14)	0.0975 (18)	−0.0299 (12)	0.0403 (14)	−0.0178 (13)
C5	0.0670 (12)	0.0496 (10)	0.0595 (11)	−0.0038 (9)	0.0225 (10)	0.0045 (9)
C6	0.0354 (8)	0.0473 (9)	0.0479 (9)	−0.0026 (7)	0.0152 (7)	−0.0100 (7)
C7	0.0383 (8)	0.0501 (9)	0.0433 (9)	0.0021 (7)	0.0123 (7)	−0.0041 (7)
C8	0.0431 (9)	0.0499 (10)	0.0466 (9)	−0.0039 (8)	0.0080 (7)	−0.0081 (8)
C9	0.0516 (10)	0.0447 (9)	0.0482 (10)	0.0061 (8)	0.0131 (8)	−0.0054 (8)
C10	0.0434 (9)	0.0477 (9)	0.0479 (9)	0.0082 (7)	0.0157 (7)	0.0030 (7)
C11	0.0407 (8)	0.0412 (9)	0.0425 (9)	0.0005 (7)	0.0134 (7)	0.0047 (7)
C12	0.0423 (9)	0.0431 (9)	0.0459 (9)	−0.0014 (7)	0.0133 (7)	−0.0063 (7)
C13	0.0436 (9)	0.0453 (9)	0.0527 (10)	−0.0013 (7)	0.0175 (8)	−0.0007 (8)

### Geometric parameters (Å, °)

**Table:**

O1—C7	1.3814 (18)	C5—H5A	0.9300
O1—C6	1.3930 (18)	C7—C12	1.379 (2)
N1—C13	1.266 (2)	C7—C8	1.381 (2)
N1—N1^i^	1.410 (3)	C8—C9	1.381 (2)
C1—C6	1.359 (2)	C8—H8A	0.9300
C1—C2	1.374 (3)	C9—C10	1.372 (2)
C1—H1B	0.9300	C9—H9A	0.9300
C2—C3	1.364 (3)	C10—C11	1.398 (2)
C2—H2A	0.9300	C10—H10A	0.9300
C3—C4	1.365 (3)	C11—C12	1.388 (2)
C3—H3A	0.9300	C11—C13	1.459 (2)
C4—C5	1.371 (3)	C12—H12A	0.9300
C4—H4A	0.9300	C13—H13A	0.9300
C5—C6	1.362 (2)		
			
C7—O1—C6	118.43 (12)	C12—C7—O1	115.75 (14)
C13—N1—N1^i^	112.25 (17)	C8—C7—O1	123.66 (14)
C6—C1—C2	118.82 (18)	C9—C8—C7	119.21 (15)
C6—C1—H1B	120.6	C9—C8—H8A	120.4
C2—C1—H1B	120.6	C7—C8—H8A	120.4
C3—C2—C1	120.4 (2)	C10—C9—C8	121.21 (15)
C3—C2—H2A	119.8	C10—C9—H9A	119.4
C1—C2—H2A	119.8	C8—C9—H9A	119.4
C2—C3—C4	119.92 (19)	C9—C10—C11	119.53 (15)
C2—C3—H3A	120.0	C9—C10—H10A	120.2
C4—C3—H3A	120.0	C11—C10—H10A	120.2
C3—C4—C5	120.2 (2)	C12—C11—C10	119.42 (14)
C3—C4—H4A	119.9	C12—C11—C13	119.11 (14)
C5—C4—H4A	119.9	C10—C11—C13	121.47 (14)
C6—C5—C4	119.14 (19)	C7—C12—C11	120.06 (15)
C6—C5—H5A	120.4	C7—C12—H12A	120.0
C4—C5—H5A	120.4	C11—C12—H12A	120.0
C1—C6—C5	121.55 (17)	N1—C13—C11	121.53 (15)
C1—C6—O1	118.67 (15)	N1—C13—H13A	119.2
C5—C6—O1	119.58 (16)	C11—C13—H13A	119.2
C12—C7—C8	120.56 (15)		
			
C6—C1—C2—C3	−0.5 (3)	O1—C7—C8—C9	178.76 (16)
C1—C2—C3—C4	0.2 (3)	C7—C8—C9—C10	0.1 (3)
C2—C3—C4—C5	0.1 (3)	C8—C9—C10—C11	−0.6 (2)
C3—C4—C5—C6	−0.2 (3)	C9—C10—C11—C12	0.3 (2)
C2—C1—C6—C5	0.4 (3)	C9—C10—C11—C13	−179.48 (15)
C2—C1—C6—O1	175.27 (16)	C8—C7—C12—C11	−1.1 (3)
C4—C5—C6—C1	0.0 (3)	O1—C7—C12—C11	−179.24 (14)
C4—C5—C6—O1	−174.87 (16)	C10—C11—C12—C7	0.5 (2)
C7—O1—C6—C1	88.2 (2)	C13—C11—C12—C7	−179.68 (14)
C7—O1—C6—C5	−96.80 (19)	N1^i^—N1—C13—C11	−179.40 (15)
C6—O1—C7—C12	−163.42 (14)	C12—C11—C13—N1	175.49 (16)
C6—O1—C7—C8	18.5 (2)	C10—C11—C13—N1	−4.7 (3)
C12—C7—C8—C9	0.7 (3)		

Symmetry codes: (i) −*x*, −*y*+1, −*z*.

### Hydrogen-bond geometry (Å, °)

**Table:**

*Cg* is the centroid of the C7–C12 benzylidene ring.

**Table:**

*D*—H···*A*	*D*—H	H···*A*	*D*···*A*	*D*—H···*A*
C5—H5A···Cg^ii^	0.93	2.68	3.5947 (17)	167

Symmetry codes: (ii) −*x*+1/2, −*y*+1/2, −*z*.
